# Hypothermia in cardiogenic shock

**DOI:** 10.1186/cc11279

**Published:** 2012-06-07

**Authors:** Jesper van der Pals

**Affiliations:** 1 Department of Cardiology, Lund University, Skane University Hospital, Sweden

## 

Cardiogenic shock is a state of inadequate systemic tissue perfusion, despite adequate left ventricular filling pressure. It is caused by extensive myocardial damage and appears to be aggravated by a systemic inflammatory response [1-4]. The result is hypotension with metabolic acidosis and often a fatal outcome. The condition affects approximately 5% of the patients with myocardial infarction, and carries a dismal prognosis if it prevails after reperfusion.

Therapeutic hypothermia has several properties of potential benefit in cardiogenic shock: Experiments with isolated myofibrils, papillary muscles and cross-circulated hearts have demonstrated that mild hypothermia increases myocardial contractility [5-7]. In the *in vivo *heart, mild hypothermia has been found to increase stroke volume and cardiac output [6,8].

The increase in contractility is considered to be mediated by an increased myofilament sensitivity to existing Ca^2+^, without a corresponding increase in myocardial oxygen consumption [9]. Moreover, hypothermia reduces the metabolic rate with 5 to 7%/°C [10,11], thereby reducing the demand on the circulation from the peripheral tissues. In an experimental setting, it also has the ability to reduce infarct size if applied prior to reperfusion [12,13].

In dog-based and porcine-based models of cardiogenic shock secondary to ischemia, therapeutic hypothermia has improved hemodynamic and metabolic parameters, and reduced mortality [14,15]. No randomized controlled trials of therapeutic hypothermia in cardiogenic shock in humans exist, but case series indicate that the effects observed in animal experiments can be reproduced [16-19].

In conclusion, therapeutic hypothermia is a promising treatment option for patients in cardiogenic shock that warrants further investigation.
